# Simultaneous Analysis of Nicarbazin, Diclazuril, Toltrazuril, and Its Two Metabolites in Chicken Muscle and Eggs by In-Syringe Dispersive Solid-Phase Filter Clean-Up Followed by Liquid Chromatography–Tandem Mass Spectrometry

**DOI:** 10.3390/foods13050754

**Published:** 2024-02-29

**Authors:** Yuxin An, Xiaodong Pan, Zengxuan Cai, Meijia Xu, Dingguo Jiang, Xiaomin Xu

**Affiliations:** 1 Zhejiang Provincial Center for Disease Control and Prevention, Hangzhou 310051, China; yxan@cdc.zj.cn (Y.A.); xdpan@cdc.zj.cn (X.P.); zxcai@cdc.zj.cn (Z.C.); mjxu@cdc.zj.cn (M.X.); 2 NHC Key Laboratory of Food Safety Risk Assessment, China National Center for Food Safety Risk Assessment, Beijing 100026, China

**Keywords:** anticoccidial drugs, eggs, chicken muscle, liquid chromatography, tandem mass spectrometry, in-syringe dispersive solid-phase clean-up

## Abstract

Nicarbazin (NICA) and triazine anticoccidial drugs (diclazuril (DIZ) and toltrazuril (TOZ)) are the primary strategy for preventing and treating coccidiosis. To prevent the development of drug resistance and mitigate the potential chronic toxicity to humans resulting from prolonged exposure, a liquid chromatography–tandem mass spectrometry method with high reliability and sensitivity was developed to determine NICA, DIZ, TOZ, and its two metabolites in chicken muscle and eggs. Upon establishing the extraction conditions involving 10 mL of acetonitrile and 10 min of sonication, in-syringe dispersive solid-phase extraction with silica was performed in combination with n-hexane clean-up. The selection of isotope peaks of precursor ions and low-mass range scanning allowed the two transitions for the quantification of all compounds. The limits of detection for DIZ and NICA were both 0.1 μg/kg, and for TOZ and metabolites, they were 0.3 μg/kg; the limits of quantitation were 0.3 and 1 μg/kg, respectively. The linear range was 0.25–50 ng/mL with a correlation coefficient r > 0.999. The average recoveries at three spiking levels in muscle and eggs were 90.1–105.2% and 94.0–103.7% with the relative standard deviations of 3.0–8.1% and 3.1–14.4%, respectively. The precision, accuracy, and stability were evaluated by three quality control samples.

## 1. Introduction

Coccidiosis is a parasitic disease caused by protozoan parasites of the apicomplexan genus *Eimeria*. It damages the intestines and prevents nutrient absorption, severely impairing the health and productivity of poultry, including extensive diarrhea, decreased feed intake and water consumption, reduced weight gain, lowered egg production, and high mortality rates [1,2]. In the currently widespread intensive farming system, it has emerged as the number one disease afflicting poultry flocks worldwide, owing to the rapid, exponential spread of the parasites through feces [3,4]. Although the development and utilization of live anticoccidial vaccines have achieved certain levels of success, the primary method for preventing and managing coccidiosis continues to be chemoprophylaxis using anticoccidial drugs [5]. The economic impact of coccidiosis in modern commercial poultry production is substantial. Nearly all commercial poultry farms regularly use anticoccidial drugs as feed additives for pullets and broiler chickens to mitigate this impact [6].

**Figure 1:**
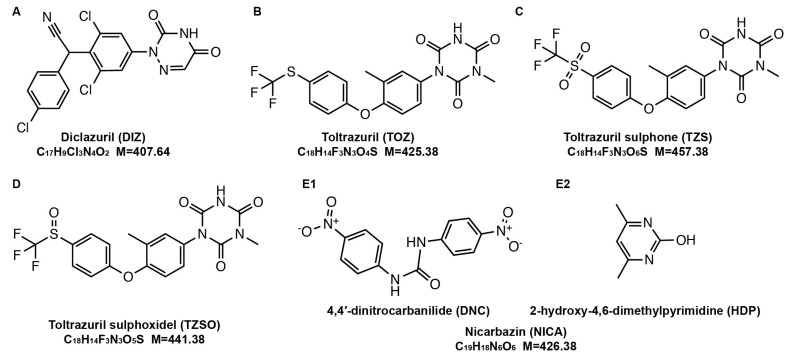
Molecular structures of five coccidiostats: (**A**) diclazuril (DIZ); (**B**) toltrazuril (TOZ); (**C**) toltrazuril sulphone (TZS); (**D**) toltrazuril sulphoxide (TZSO); (**E1**) 4,4′-dinitrocarbanilide (DNC); (**E2**) 2-hydroxy-4,6-dimethylpyrimidine (HDP).

Nicarbazin (NICA) and triazine anticoccidial drugs (diclazuril (DIZ) and toltrazuril (TOZ)) are the commonly used chemical coccidiostats. NICA refers to the combination of 4,4′-dinitrocarbanilide (DNC) and 2-hydroxy-4,6-dimethylpyrimidine (HDP) in a 1:1 molar ratio. DNC is commonly employed as the marker for the residue of NICA in animal food [7]. The structures of these drugs are shown in Figure 1. Under Regulation 1831/2003/EC, coccidiostats are currently authorized as feed additives [8]. These coccidiostats can be used at specified concentrations and within certain timeframes for broilers and young chickens, but their usage is not permitted for laying hens. For muscle, the following maximum residue limits (MRLs): 500 μg/kg for DIZ, 100 μg/kg for TOZ, and 200 μg/kg for NICA were set [9]. TOZ is absorbed via the gastrointestinal tract and metabolized into the biologically active forms toltrazuril sulphoxide (TZSO) and toltrazuril sulphone (TZS) [10]. Over the past few decades, global attention has increasingly focused on food safety as a matter of paramount importance. As for coccidiostats, a suspensory period before animal slaughtering was asked for metabolizing and eliminating from the organism. Even so, there are still many cases where drugs have been found in some animal products [11], resulting from a failure to fully follow recommended practices, a relatively slow depletion, or from cross-contamination during feed production and storage [12]. The drug and metabolite residues persist in the environment, exhibiting a relatively high phytotoxicity. This could potentially lead to the emergence of resistant strains and trigger allergic reactions in susceptible individuals [13]. In addition, the long-term effects of the habitual ingestion of low-dose veterinary drug mixtures coming from the consumption of products and drinking water supplies are difficult to predict [14]. Hence, a reliable, selective, and sensitive analytical method is required for the analysis of compounds at trace level in food samples, especially for the drugs that cannot be present in eggs.

Among the technique for analyzing drug residues in animal-derived food products, liquid chromatography coupled with tandem mass spectrometry (LC-MS/MS) has been proven to have high sensitivity and reliability [15] (Table 1). Matabudul et al. [16] described a method using a silica cartridge for the detection of NICA in animal livers and eggs with a limit of quantitation (LOQ) of 2.5 μg/kg. For the determination approach using LC-MS/MS, at least one precursor and two product ions are required for fulfilling the legislation in terms of quantitative and confirmatory analysis [17,18]. It is challenging for TOZ and its two metabolites (TZSO and TZS) to meet the demands. Sun et al. [9] employed gel permeation chromatography (GPC) combined with MS/MS and achieved a LOQ of 1.2 μg/kg for DIZ and TOZ, and 1.8 μg/kg for TZSO and TZS in real samples. In that report, the quantitative analysis of TOZ and its metabolites was only performed using the precursor ions, without providing sufficient identification points (IPs) for targeted compound quantification. In 2023, Rydchuk et al. performed a method utilizing ultrasonic extraction for chicken meat using acetonitrile (ACN) and a buffer solution, followed by extraction with organic solvents. The limits of detection (LOD) and LOQ for TOZ reached 1.2 and 4.1 μg/kg. However, there were still insufficient transitions available, limiting the quantitative analysis accuracy and stability. Martinez-Villalba et al. [19] developed the clean-up using a solid-phase extraction (SPE) cartridge coupled with an LC-MS/MS method under the atmospheric pressure chemical ionization mode, yielding the ions [M-CF3]^−^ or [M-CHF3]^•−^ as the precursor ions for acquiring sufficient fragment ion information. Consequently, the signal-to-noise ratio (S/N) for the analysis of complex samples was improved, and the LOD for TOZ and TZS reached 0.5 μg/kg; while the LOD for TZSO was 5 μg/kg, as only the transition of precursor > precursor could be used.

To fulfill the demand for efficient, highly sensitive, and accurate detection of compounds like NICA, DIZ, TOZ, TZSO, and TZS in food-derived samples, our work here presents a method that utilizes an in-syringe dispersive solid-phase filter extraction clean-up followed by an LC-MS/MS analysis.

## 2. Materials and Methods

### 2.1. Chemicals and Reagents

All reagents and solvents were of analytical grade unless specified.

DIZ, TOZ, and DIZ-^13^C_3_^15^N_2_ were purchased from Dr. Ehrenstorfer (Augsburg, Germany). TZS, TZSO, TOZ-D_3_, and DNC were provided by BePure (Beijing, China). DNC-D_8_ was supplied by Witega (Berlin, Germany). All standards were at a 99.5% minimum purity. Dimethylformamide (DMF) was obtained from Shuanglin Huagong (Hangzhou, China). Sodium chloride and HPLC grades of methanol, formic acid, ACN, hexane (95%) were purchased from Thermo Fisher Scientific (Shanghai, China). Silica, C18, and primary secondary amine (PSA) were purchased from Agela Technologies (Tianjin, China). Envi-carb powder (Carb) was bought from Merck (Darmstadt, Germany). Deionized water was obtained by a Milli-Q system (Milford, MA, USA).

### 2.2. Standard Solutions’ Preparation

Single standard stock solutions (DIZ, TOZ, TZSO, TZS, DNC) and single internal standard (IS) stock solutions (DIZ-^13^C_3_^15^N_2_, TOZ-D_3_, DNC-D_8_) were prepared in DMF at a concentration of 100 mg/L each. A mixed standard or IS solution with a concentration of 10 mg/L was prepared in DMF. A mixed standard or IS working solution with a concentration of 1 mg/L was prepared in methanol. All solutions were stored at −18 °C for 6 months. A seven-point (0.25, 0.5, 1, 2.5, 5, 20, and 50 ng/mL) standard series was prepared for making a calibration curve by diluting the standard working solution in ACN–water (50/50, *v*/*v*), with the concentration of the IS at 5 ng/mL for each point.

### 2.3. Sample Preparation

#### 2.3.1. Chicken Muscle and Egg Samples Preparation

Minced chicken muscle or a shell-removed egg was subjected to high-speed blending for thorough mixing. After the determination of DIZ, TOZ, TZSO, TZS, and DNC by the developed method, the analytes-free sample was used as the blank sample. The assessment of accuracy, precision, and stability was accomplished by quality control (QC) samples. The standard solution was spiked into the pooled blank matrix to prepare QC samples, with spiking concentrations of 10 μg/kg in eggs and 10 μg/kg, 200 μg/kg, and 500 μg/kg in chicken muscle. The samples were stored at −18 °C and tested as soon as possible.

#### 2.3.2. Sample Analysis

An aliquot of 5 g was weighed into a 50 mL centrifuge tubes. Then, 100 μL of a 1 μg/mL IS solution was added to the sample. Subsequently, 2 mL of water (not required for eggs) and 10 mL of ACN were added. The mixture was vortexed for 5 min, followed by 20 min of sonication extraction. Next, approximately 2 g of sodium chloride was introduced into the mixture, which was then vortexed for 2 min and centrifuged at 4000 rpm for 5 min. A 1 mL volume of the supernatant was transferred to a syringe containing 50 mg of silica and a 0.22 µm PTFE filter. Subsequently, the plunger rod was connected, and the solution was pushed through the material and PTFE filter. The elution was collected at a rate of about 1 drop/s. Following this, 0.5 mL of water and 0.5 mL of n-hexane were added to 0.5 mL of eluent. The mixture was vortexed for 10 s and centrifuged at 4000 rpm for 2 min. The upper layer of n-hexane was discarded, and the sample was prepared for the LC-MS/MS analysis.

#### 2.3.3. Optimization for Sample Extraction Conditions

The volume of ACN for extraction was optimized based on the QC sample at a concentration of 10 μg/kg for egg and muscle samples. Each 5 g of the QC sample was extracted with 5, 10, or 15 mL of ACN, respectively. The remaining sample analysis procedure followed that outlined in Section 2.3.2.

The total duration of sonication extraction was determined as well. Similarly, sonication extraction was performed for 5, 10, and 15 min, respectively. The remaining sample analysis procedure followed that outlined in Section 2.3.2. Three repetitions were performed for each condition.

#### 2.3.4. Recovery in Different Clean-Up Material 

An aliquot of 1 mL of ACN was spiked with 10 μL of standard solution (1 μg/mL). After being mixed, the mixture was transferred to a syringe containing a 0.22 µm PTFE filter and various dispersive solid-phase extraction (DSPE) materials (50 mg of C18, PSA, Carb, or silica). Subsequently, the plunger rod was connected, and the solution was pushed through the material and PTFE filter. The filtrate was then subjected to the LC-MS/MS analysis. This process was repeated three times. The recoveries of analytes were calculated as 100 × (peak area in the filtrate/peak area in pure solvent).

An aliquot of 10 μL of standard solution (1 μg/mL) was spiked into 1 mL of ACN and processed following the procedure outlined in Section 2.3.2. A different amount of silica (50/100/150 mg) was used than for the DSPE material. Three repetitions were performed for each dosage.

#### 2.3.5. Matrix Effects after DSPE Material Clean-Up

An aliquot of 5 g of chicken muscle or egg blank sample was processed following the procedure outlined in Section 2.3.2, with the exception that no IS was added. Each 1 mL of supernatant was then transferred to a syringe containing a 0.22 µm PTFE filter and 50 mg of C18 or silica. Subsequently, the filtrate was added to 0.5 mL of water, 0.5 mL of n-hexane, and 10 μL of mixed standard working solution (1 μg/mL). The down layer was subjected to the LC-MS/MS analysis. Three repetitions were performed. Matrix effects were determined as 100 × (peak area in matrix/peak area in pure solvent). Values close to 100% indicated no matrix effects, while values below or above 100% indicated matrix suppression or enhancement effects.

#### 2.3.6. Method Recovery

Method recoveries were evaluated at the levels of 1 μg/kg, 10 μg/kg, 20 μg/kg in eggs and 1 μg/kg, 100 μg/kg, 200 μg/kg in chicken muscle. Each 5 μL of 1 mg/L, 10 mg/L, 20 mg/L, 100 mg/L, and 200 mg/L of standard solution was spiked into 5 g blank sample. The sample was vortex-mixed for 5 min and spiked with 100 μL of IS (1 mg/L). The remaining steps were the same as Section 2.3.2. Six repetitions were performed for each spiking level.

#### 2.3.7. Accuracy and Precision

Accuracy and precision were assessed using QC samples at levels of 10 μg/kg in eggs and 200 μg/kg and 500 μg/kg in chicken muscle. Each 5 g QC sample was processed following the procedure outlined in Section 2.3.2. Six repetitions in the same day and three repetitions in three days, respectively, were performed for intradays and interdays’ accuracy and precision.

### 2.4. Instrumentation

LC-MS/MS with a negative electrospray ionization (ESI^−^) source (Shimadzu 8060 LC-MS, Kyoto, Japan) was employed. Data acquisition and analysis were conducted using LabSolutions version 5.97 SP1. For the LC separation, an XBridge™ BEH C18 column (Waters, Milford, MA, USA, 2.1 mm id × 10 cm, 2.5 µm) was utilized, maintained at 40 °C with a flow rate of 0.35 mL/min. The mobile phase consisted of 0.03% (*v*/*v*) formic acid (Channel A) and methanol (Channel B). The gradient elution proceeded as follows: from 45% to 90% in 3 min, maintained for 2 min, then returned to 45% B in 0.5 min and maintained for 3.5 min. The mass spectrometer parameters included an interface temperature of 300 °C, a desolvent line temperature of 250 °C, and a heat block temperature of 400 °C. Nebulizing gas and drying gas were nitrogen flowing at rates of 3 L/min and 10 L/min, respectively. Heating gas was provided by compressed air at a flow rate of 10 L/min, and argon was employed as the CID gas at 270 kPa. The interface voltage was adjusted to 4.5 kV. Multiple reaction monitoring (MRM) mode was utilized, and detailed mass spectrometer parameters for the acquisition of each analyte are provided in Table 2.

## 3. Results and Discussion

### 3.1. Extraction and Clean-Up

The primary objective of this study was to establish a rapid and effective quantitation method for DIZ, TOZ, TZSO, TZS, and NICA in chicken muscle and eggs. In this study, we used an in-syringe DSPE, a widely utilized technique for rapid sample pretreatment [21,22]. The whole procedure is easy to operate and fast, involving extraction with ACN, the addition of NaCl to remove large amounts of protein from the samples, followed by the addition of a suitable sorbent and further n-hexane to eliminate both polar and fat-soluble interferences in the samples. Compared to the SPE clean-up method used in previous studies which often required several hours to process, this method only involves three solution extractions and can be completed within 30 min. The introduction of in-syringe DSPE significantly enhances sample processing throughput.

**Figure 2:**
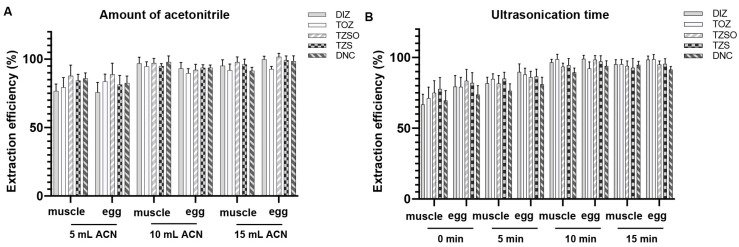
Extraction efficiency of analytes in different volumes of ACN (**A**) and different times of ultrasonication (**B**) for chicken muscle and eggs (*n* = 3).

In previous reports, ACN was employed as a favorable extraction solvent for these compounds [9,23]. Additionally, ACN offers the advantage of precipitating proteins in the samples. According to the operation in Section 2.3.3, the recoveries with different extraction volumes are depicted in Figure 2A. Upon increasing the solvent volume for extraction to 10 mL, a marked improvement in the extraction efficiency of each compound was observed. Next, an optimization of the ultrasonication time was conducted, and it was established that 10 min essentially ensured the complete extraction of all components (Figure 2B).

**Figure 3:**
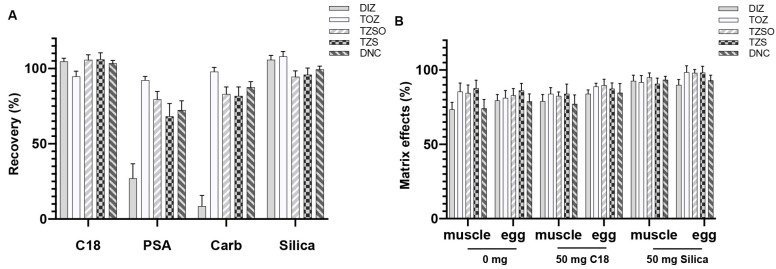
Recoveries of analytes in solvent (**A**) and matrix effects in sample (**B**) after in-syringe DSPE clean-up using different materials (*n* = 3).

**Figure 4:**
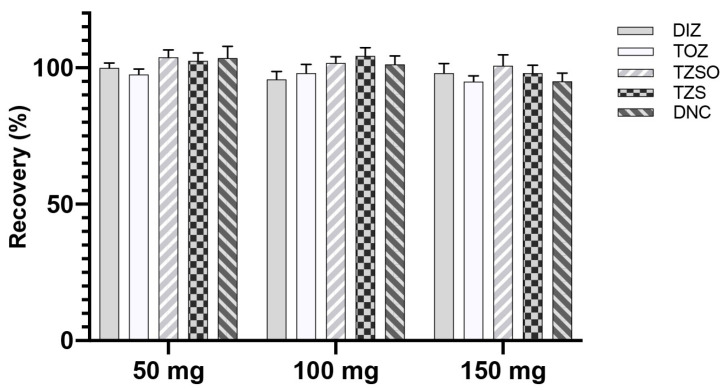
Recoveries of analytes in solvent after in-syringe DSPE clean-up using different amounts of silica (*n* = 3).

According to the operation in Section 2.3.4, the recoveries of analytes after being cleaned with different DSPE materials are shown in Figure 3A. Low recoveries were found for DIZ when treated with PSA and Carb; and the recoveries of TOZ, TZSO, and TZS exhibited significant decreases when compared to the use of C18 or silica, which makes them promising candidates for the DSPE clean-up of analytes. Consequently, we compared the matrix effects in the sample after applying C18 and silica. The utilization of C18 resulted in recoveries ranging from 75.5% to 89.8% in chicken muscle and eggs, while silica achieved recoveries ranging from 90.1% to 98.1% (Figure 3B). The amount of silica used for clean-up was further examined. Increasing the amount of silica from 50 mg to 150 mg did not significantly improve recoveries (Figure 4). In summary, 50 mg of silica is a suitable choice for DSPE clean-up.

### 3.2. Selection of Mass Spectrometry Conditions

**Figure 5:**
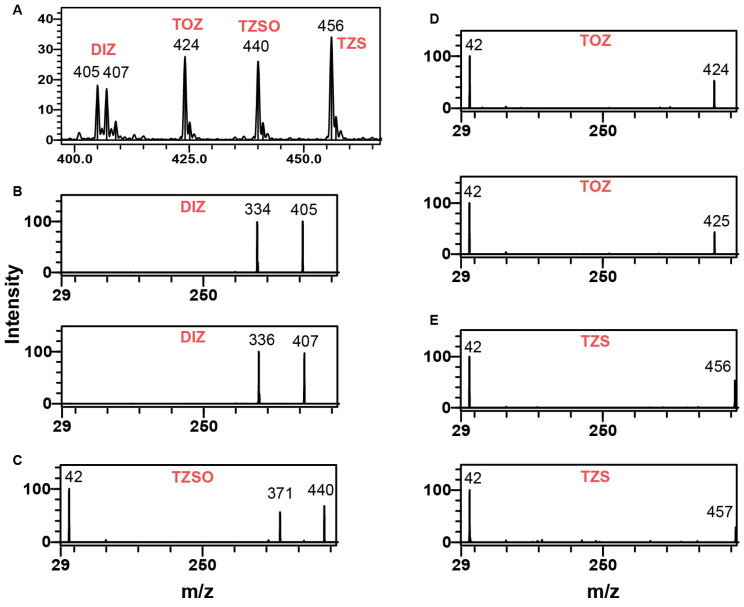
Mass spectra of DIZ, TOZ, TZSO, and TZS. Full-scan spectrum (**A**) and product ion scan spectra of DIZ (**B**), TZSO (**C**), TOZ (**D**), and TZS (**E**). X axis is mass divided by charge number (*m*/*z*) for the ions, y axis represents signal intensity of the ions.

According to the European Commission Guideline for the identification and quantification of organic residues and contaminants, either one precursor and two product ions or two precursor ions, each with one product ion, are required [24]. As shown in the full-scan mass spectrum (Figure 5A), the natural isotope peaks of DIZ, TOZ, TZSO, and TZS molecular ions are clearly observable. Furthermore, the relative abundance of these isotopic ions is near 20%, enabling the use of the natural isotope as the second precursor ion. Thus, in the case of DIZ, five IPs could be achieved by selecting two precursors and corresponding product ions (Figure 5B). However, for TOZ and its two metabolites, prior studies indicated that the deprotonated molecule ([M-H]^−^) was the sole observable ion in the full-scan mass spectrum, without yielding suitable fragment ions [9,12,19,20]. In the lower mass range, we detected a fragment ion with an m/z of 42 for TOZ, TZSO, and TZS (Figure 5C–E).

**Figure 6:**
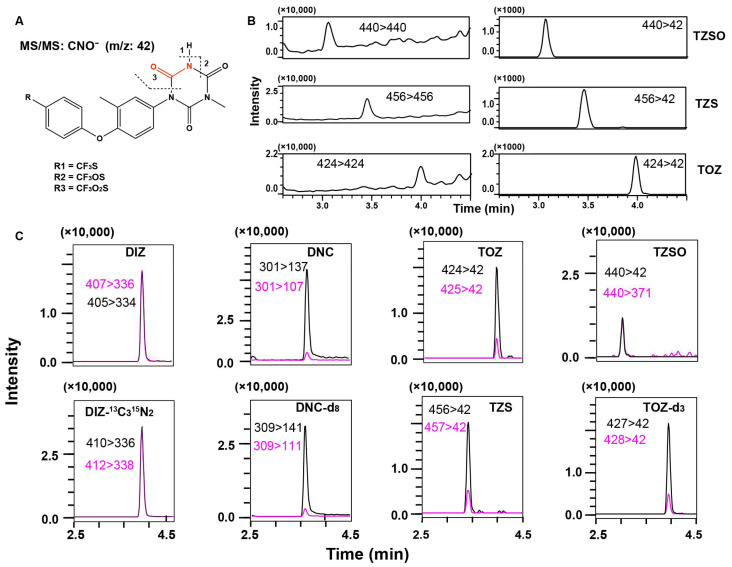
Fragment ions of TOZ, TZSO, and TZS in triple quadrupole mass spectrometry (**A**). MRM chromatograms comparisons of TZSO, TZS, and TOZ in chicken eggs with single MS (left) and new parent/product ion pairs (right), respectively (**B**). MRM chromatograms of all analytes and their deuterated internal standards with concentrations of 10 μg/kg in eggs (**C**).

The fragments of the corresponding characteristic ions in MS/MS are depicted in Figure 6A. According to the operation in Section 2.4, chromatograms acquired using this transition in the complex matrix exhibited significantly improved S/N compared to the single MS (Figure 6B). Moreover, the natural isotope ions (precursor ions, *m*/*z* 425 for TOZ and 457 for TZS) and the corresponding fragment ion could be used as the other transition for meeting the MS/MS identification criteria effectively. Therefore, when an adequate number of precursor/product ion pairs cannot be provided by the compounds themselves, consideration can be given to natural isotope peaks of the precursor ions [25]. So far, all analytes possess at least four IPs for satisfying the identification requirements of MS/MS (Figure 6C).

### 3.3. Method Validation

#### 3.3.1. Linear Range and LODs

The method was validated prior to its application in the sample analysis. The linear range was 0.25 ng/mL–50 ng/mL, with the correlation coefficient (r) exceeding 0.999 for each analyte (Table 3). The LOD was based on an S/N of three in the sample matrix at a concentration near LOD. The values were 0.1 μg/kg for DIZ and NICA, and 0.3 μg/kg for TOZ, TZSO, and TZS in chicken muscle and eggs. The LOQ was set at 0.3 and 1 μg/kg, respectively, based on an S/N of 10. Since their usage is not permitted for laying hens, coccidiostats could not be detected in eggs. Compared to the LOD achieved by the organic solvent extraction method (2 μg/kg for DIZ and DNC; 1.2, 1.6, and 3.2 μg/kg for TOZ, TZS, TZSO)^7^, this approach is a significant improvement. 

#### 3.3.2. Recovery and Relative Standard Deviation

Taking into account the LOQ, the medium and high points within the linear range, as well as the MRL, the spiking levels were established at 1, 10, 20, 100, and 200 μg/kg with six parallel measurements. The results are presented in Table 4. The average recoveries for eggs ranged from 90.1% to 105.2%, with relative standard deviations (RSDs) of 3.0% to 8.1%. The average recoveries in muscle were 94.0–103.7% with RSDs of 3.1–11.4%.

#### 3.3.3. Accuracy and Precision

The optimized method was assessed by analyzing QC samples, as detailed in Section 2.3.7. Three samples were prepared, including those with concentrations of 10 μg/kg in eggs and 200 μg/kg and 500 μg/kg in muscle. Six parallel measurements were determined to assess the intradays’ recovery and precision. Furthermore, two additional repetitions for each sample were analyzed on five different days to evaluate the interdays’ accuracy and precision. As shown in Table 5, the average recoveries of intradays were 90.5–99.1% with RSDs of 2.2–6.9%, and the average recoveries of interdays were 89.9–105.3% with RSDs of 3.8–7.6%. This indicated that this method for the simultaneous analysis of DIZ, NICA, TOZ and its two metabolites in eggs and chicken muscle has superior accuracy, repeatability, and reproducibility.

### 3.4. Real Sample Analysis

The developed analytical method was utilized to analyze 83 chicken muscle samples collected from the market. DNC was found to have the highest detection rate (16.87%), with a concentration range of 0.6–82.5 μg/kg. DIZ was detected in 11 samples (13.25%), ranging from 0.2 to 27.3 μg/kg, and TZS was detected in 8 samples (9.64%), ranging from 2.0 to 134 μg/kg. In the sample with a TZS content of 134 μg/kg, trace amounts of TOZ and TZSO were detected, with concentrations of 1.8 μg/kg and 2 μg/kg, respectively. Among these, one sample exceeded the MRL for TOZ in chicken muscle (calculated as TZS), while the levels of other drugs were all within the MRLs. Out of the 193 collected egg samples, DNC had a detection frequency of 2.07%, with a concentration range of 2.9–121 μg/kg, and DIZ was detected in 2.07% of samples, ranging from 0.2 to 27.3 μg/kg. TZS was only detected in one sample with a concentration of 4.0 μg/kg, while TOZ and TZSO were undetectable. It could be found that highly sensitive detection of TZS and DNC could be achieved in chicken muscle and egg samples. Both EU and Chinese regulatory standards employ TZS and DNC as the respective residue markers for monitoring TOZ and NICA, contributing to the effective regulation of such drugs.

## 4. Conclusions

In addressing the challenge of identifying residues of TOZ and its metabolites using LC-MS/MS, where only precursor ions are available for identification, integrated MS conditions were established. This involved confirming fragment ions through low-mass range scanning and further utilizing the natural isotope peaks of precursor ions along with their corresponding fragments as IPs, significantly enhancing the precision and sensitivity of detection. Further combined with the optimal extraction and in-syringe DSPE clean-up condition, a simultaneous analysis method of NICA, DIZ, TOZ, and its two metabolites was established. This method has superior efficiency and accuracy and would be helpful in monitoring the use of anticoccidial drugs, ensuring food safety and mitigating potential health risks. In addition, from the results of the real sample analysis, it can be observed that using TZS and DNC as the residue markers for TOZ and NICA, respectively, is feasible.

## Figures and Tables

**Table 1:** Extraction method, clean-up method, MRM parameters, LOD, and LOQ in previous works for identification of anticoccidial drugs in food samples.

Analytes	Sample	Extraction Method	Clean-Up Method	*m/z*	LOD	LOQ
NICA [16]	Egg	H_2_O; ACN	Silica SPE	301/137 301/107	1	2.5
DIZ; TOZ; TZS; TZSO [9]	Muscle; egg	Ethylacetate	GPC	DIZ: 404/334 TOZ: 424/424 * TZS: 456/456 * TZSO: 440/371	0.4 for DIZ and TOZ; 0.6 for TZSO and TZS	1.2 for DIZ and TOZ; 1.8 for TZSO and TZS
TOZ; TZS; TZSO [19]	Meat product	ACN	Silica SPE	APCI TOZ: 356.1/256.0 356.1/129.9 TZSO: 371.1/371.1 * 371.1/271.1 TZS: 388.1/288.0 388.1/248.0	0.5 for TOZ and TZS 5 for TZSO	/
DIZ; NICA [8]	Chicken liver	ACN	HLB SPE	DIZ: 407/336 405/334 NICA: 301/137 301/107	1 for DIZ 2 for NICA	3 for DIZ 5 for NICA
DIZ; NICA; TOZ; TZS; TZSO [20]	Muscle; egg	ACN	/	NICA: 301.1/107.0 301.1/136.9 TOZ: 424.2/424.2 * TZS: 456.2/456.2 * TZSO: 440.1/440.1 * DIZ: 405.0/333.9 407.0/335.9	/	1
DIZ; NICA; TOZ; TZS; TZSO [7]	Poultry liver; muscle	Phosphate-citrate buffer (pH 9.7); ACN	Ethylacetate; dichloromethane; hexane	NICA: 301.1/106.9 301.1/136.0 TOZ: 424.0/424.0 * 424.0/41.8 TZS: 456.2/456.2 * 456.2/41.9 TZSO: 440.2/371.2 440.2/41.9 DIZ: 405.0/334.0 405.0/301.0 407.0/336.0	2 for NICA 2 for DIZ 1.2 for TOZ 1.6 for TZS 3.2 for TZSO	6.6 for NICA 6.6 for DIZ 4.1 for TOZ 5.4 for TZS 10.5 for TZSO

* The transition of precursor > precursor was used.

**Table 2:** MRM conditions for anticoccidial drugs and IS.

Compound	*m/z*, Precursor Ion	*m/z*, Product Ion	Collision Energy (eV)	IS Group
DIZ	405 407	334 336	18 18	DIZ-^13^C_3_^15^N_2_
TOZ	424 425	42 42	20 20	TOZ-D_3_
TZSO	440 440	42 371	15 20	TOZ-D_3_
TZS	456 457	42 42	20 20	TOZ-D_3_
DNC	301 301	137 107	15 33	DNC-D_8_
DIZ-^13^C_3_^15^N_2_	410 412	336 338	18 18	/
TOZ-D_3_	427 428	42 42	20 20	/
DNC-D_8_	309 309	141 111	15 33	/

**Table 3:** Linearity, correlation coefficients, LODs, and LOQs of the developed method of analytes.

Analytes	Linear Range (μg/kg)	Correlation Coefficient R	LOD (μg/kg)	LOQ (μg/kg)
DIZ	1–200	0.999	0.1	0.3
DNC	1–200	0.997	0.1	0.3
TOZ	1–200	0.998	0.3	1
TZSO	1–200	0.994	0.3	1
TZS	1–200	0.998	0.3	1

**Table 4:** Average recoveries and RSDs of analytes at various spiking levels (*n* = 6) in eggs and chicken muscle.

Analytes	Matrix	Eggs			Muscle		
	Spiked (μg/kg)	1	10	20	1	100	200
DIZ	Recovery (%)	92.1	94.2	92.1	103.7	98.3	94.8
	RSD (%)	3.3	5.1	3.5	8.2	6.1	3.1
TOZ	Recovery (%)	94.7	97.9	95.3	102.9	94.8	97.7
	RSD (%)	4.1	8.1	4.1	5.8	6.5	7.9
TZSO	Recovery (%)	92.6	105.2	93.5	102.5	94.9	96.5
	RSD (%)	3.1	5.4	3.0	14.4	4.1	7.4
TZS	Recovery (%)	90.1	96.8	95.3	101.2	94.6	94.6
	RSD (%)	4.9	3.6	3.4	8.6	6.1	7.2
DNC	Recovery (%)	95.4	92.6	100.2	101.1	94.0	98.2
	RSD (%)	4.0	6.2	7.4	11.4	4.4	5.4

**Table 5:** Intradays and interdays’ precision of analytes in QC samples.

Matrix	Spiked (μg/kg)	Analytes		DIZ	TOZ	TZSO	TZS	DNC
Eggs	10	Intraday	Recovery (%)	92.5	92.0	96.8	90.5	93.1
			RSD (%)	5.4	3.1	3.8	6.9	6.4
		Interday	Recovery (%)	96.0	92.0	89.9	91.6	90.7
			RSD (%)	6.2	4.1	7.6	7.6	6.0
Muscle	200	Intraday	Recovery (%)	97.4	94.6	99.1	93.8	92.9
			RSD (%)	2.4	5.4	2.9	4.1	3.1
		Interday	Recovery (%)	94.1	100.5	98.4	105.3	91.3
			RSD (%)	4.3	6.2	4.6	4.4	6.3
	500	Intraday	Recovery (%)	90.6	95.3	91.3	94.5	91.8
			RSD (%)	5.2	4.1	2.2	5.3	4.9
		Interday	Recovery (%)	99.3	90.4	92.7	102.2	98.6
			RSD (%)	3.8	5.6	4.3	4.2	5.8

## Data Availability

Data are contained within the article.
